# Is the high-risk strategy to prevent cardiovascular disease equitable? A pharmacoepidemiological cohort study

**DOI:** 10.1186/1471-2458-12-610

**Published:** 2012-08-04

**Authors:** Helle Wallach-Kildemoes, Finn Diderichsen, Allan Krasnik, Theis Lange, Morten Andersen

**Affiliations:** 1 Centre for Healthy Aging, Department of Public Health, University of Copenhagen, Øster Farimagsgade 5, Copenhagen, 1014, Denmark; 2 Social Medicine, Department of Public Health, University of Copenhagen, Øster Farimagsgade 5, Copenhagen, 1014, Denmark; 3 Health Services Research, Department of Public Health, University of Copenhagen, Øster Farimagsgade 5, Copenhagen, 1014, Denmark; 4 Biostatistics, Department of Public Health, University of Copenhagen, Øster Farimagsgade 5, Copenhagen, 1014, Denmark; 5 Centre for Pharmacoepidemiology, Karolinska Institutet, Stockholm, SE-171 77, Sweden; 6 Research Unit for General Practice, Institute of Public Health, University of Southern Denmark, B. Winsløws Vej 9A, Odense, 5000, Denmark

**Keywords:** Statins to prevent cardiovascular disease, The high-risk strategy, Socioeconomic gradient, Horizontal equity

## Abstract

**Background:**

Statins are increasingly prescribed to prevent cardiovascular disease (CVD) in asymptomatic individuals. Yet, it is unknown whether those at higher CVD risk – i.e. individuals in lower socio-economic position (SEP) – are adequately reached by this high-risk strategy. We aimed to examine whether the Danish implementation of the strategy to prevent cardiovascular disease (CVD) by initiating statin (HMG-CoA reductase inhibitor) therapy in high-risk individuals is equitable across socioeconomic groups.

**Methods:**

Design: Cohort study.

*Setting and participants:* Applying individual-level nationwide register information on socio-demographics, dispensed prescription drugs and hospital discharges, all Danish citizens aged 20+ without previous register-markers of CVD, diabetes or statin therapy were followed during 2002–2006 for first occurrence of myocardial infarction (MI) and a dispensed statin prescription (N = 3.3 mill).

*Main outcome measures:* Stratified by gender, 5-year age-groups and socioeconomic position (SEP), incidence of MI was applied as a proxy for statin need. *Need-standardized statin incidence rates* were calculated, applying MI incidence rate ratios (IRR) as need-weights to adjust for unequal needs across SEP.Horizontal equity in initiating statin therapy was tested by means of Poisson regression analysis. Applying the need-standardized statin parameters and the lowest SEP-group as reference, a need-standardized statin IRR > 1 translates into horizontal inequity favouring the higher SEP-groups.

**Results:**

MI incidence decreased with increasing SEP without a parallel trend in incidence of statin therapy. According to the regression analyses, the need-standardized statin incidence increased in men aged 40–64 by 17%, IRR 1.17 (95% CI: 1.14-1.19) with each increase in income quintile. In women the proportion was 23%, IRR 1.23 (1.16-1.29). An analogous pattern was seen applying education as SEP indicator and among subjects aged 65–84.

**Conclusion:**

The high-risk strategy to prevent CVD by initiating statin therapy seems to be inequitable, reaching primarily high-risk subjects in lower risk SEP-groups.

## Background

A steep inverse relationship between socioeconomic position (SEP) and incidence of cardiovascular disease (CVD) has consistently been shown across high-income Western countries [1-3]. The social gradient has widened over the last decades [4,5] and is to a large extent mediated by the conventional risk factors (i.e., smoking, high blood pressure and serum cholesterol) – when evaluated in absolute terms [1,6-8]. This holds also for the most important CVD component, myocardial infarction (MI) [9].

As CVD is one of the leading causes of premature death in the Western world, preventive strategies are on political agendas, all focusing on the conventional risk-factors, either through their socio-cultural determinants (population (structural) strategies) or through individual behaviour/risk-factors, such as the high-risk strategy to prevent CVD in general practice. In the high-risk strategy (often termed ‘primary prevention’), asymptomatic individuals are screened to determine the need for preventive interventions, such as antihypertensives or lipid lowering drugs (almost exclusively statins). In the present study, we focus on statins (HMG-CoA reductase inhibitors), introduced in 1994 [10] to, reduce post-MI mortality in middle-aged men with hypercholesterolemia. Following subsequent randomised clinical trials (RCTs), recommendations for statins have broadened, including now also asymptomatic individuals (i.e., without established CVD or diabetes) irrespective of lipid-levels age and gender. “The question of ‘at what lipid level to initiate treatment’ has to be replaced by ‘at what cardiovascular risk should statins be started’“[11].

The high-risk strategy has been implemented in Denmark as an opportunistic screening strategy i.e. clients who show up in the general practitioner’s (GP) office may be screened for high CVD risk for possible prescription of preventive drugs. In line with the European guidelines and the European Systematic Coronary Risk Evaluation [12], Danish GPs are recommended to use a matrix of serum lipid and blood pressure levels (stratified by age, gender and smoking status) for identifying high-risk individuals, applying an estimated 10-year risk of fatal atherosclerotic events above 5% as high risk threshold. While risk thresholds and CVD endpoints vary slightly according to country, all risk-score charts are based on the same risk-factor matrix, providing risk estimates based on data and risk-equations from historic cohort studies and RCTs [12-14].

The Danish health care system is mainly tax-financed and based on the egalitarian principle, with equal health care (access/treatment/outcome) for equal (legitimate) health care needs [15] – also called horizontal equity [16]. While authorized GP services are free, prescription drugs require patient co-payment. Based on decisions by an authority under the Ministry of Health, the actual amount of reimbursement depends on whether a particular drug is reimbursable and the actual reimbursement schedule for reimbursable drugs [17]. The current ‘need-dependent’ reimbursement schedule (introduced March 2000) has a number of reimbursement levels, the reimbursed percentage increasing stepwise with the individual’s annual drug expenditures. Reimbursement is based on the cheapest generic drug [17]. Despite near universal health care coverage in many European countries, income-related inequalities in the use of physician services have been observed [18-20]. In Denmark this holds true especially in regards to elective procedures [21] and services with co-payments, such as prescription drugs [22-24]. Yet, European health-care systems are under pressure due to increasing health care expenditures and the challenges of an ageing population [25,26], which includes shortage of GP’s partly due to the retirement of the baby-boom generation.

There is an ongoing debate about the high-risk strategy [14,27-29], encompassing allocation of scarce health care resources [28] and “The strategy of preventive medicine”, by Geoffrey Rose [30], i.e., the high-risk strategy versus the population strategy [29,31,32]. As reduction of social inequalities in health is a central goal in WHO and EU programmes [33,34], it is also being debated whether or not these strategies will reduce inequalities in CVD [35,36]. A range of studies have explored inequalities in utilisation of CVD drugs, but without explicitly taking need-determined measures into account, some focusing on regional or socioeconomic inequalities [37-40], others restricting analyses to individuals with the same medical condition [23,41]. In a study of equity in statin prescribing by GPs in the UK, the authors explore to what extent prescribing variations in different primary care trusts are associated with the frequency of CVD admissions and socio-demographic characteristics [38]. Assuming implicitly equal needs across these groups, the results of the UK study could indicate inequitable statin prescribing. Yet, inequality in health care delivery can only be interpreted as inequity if legitimate need-determined inequalities are taken into account [19,20].

In the present study, we focus on initiation of preventive statin therapy in the high-risk strategy as implemented in Denmark. Due to the social gradient in incidence of CVD we expect an increasing need for CVD preventive drugs with decreasing SEP i.e. unequal needs across socioeconomic groups. In line with other studies focusing on equity in health care delivery [22,42], we assume that equity will be met if care is provided proportionally to the need. To our knowledge no studies has explored to what extent the high-risk strategy to reduce CVD is equitable.

The aim of this study was to examine whether the Danish implementation of the strategy to prevent CVD by initiating statin therapy in high-risk individuals is equitable across socioeconomic groups; hypothesising that this high-risk strategy will not adequately reach groups with a lower SEP, characterised by having a higher risk of CVD.

## Methods

### Data source and participants

From nationwide Danish registers maintained by the National Board of Health and Statistics Denmark, we retrieved individual-level information on dispensed prescription drugs, hospital discharges, dates of death or emigration, and socioeconomic indicators. Data were linked by means of a unique encrypted person-identifier, allowing authorised researchers to follow individuals in multiple individual-level registries hosted in Statistics Denmark. Register-based studies in Denmark do not require approval by an ethics board.

A cohort corresponding to all Danish citizens aged 20+ without previous register markers of CVD, diabetes or statin treatment were followed, during 2002–2006, in the registries, for dispensed statin prescriptions and the first occurrence of MI (N = 3.3 mill). We applied two different SEP indicators: disposable family income and highest attained education (as of 2002). Table 1 shows the characteristics of the cohort of asymptomatic individuals, by gender, age and highest attainted education, demonstrating that historical information on education is poorly covered among persons older than 75.

**Table 1:** The cohort as of January 1, 2002, according to gender, age and educational level

**Gender**	**Educational Level**^**a**^	**20-39**		**40-54**		**55-64**		**65-74**		**75-84**		**85+**		**All**
		**N**	**(%)**	**N**	**(%)**	**N**	**(%)**	**N**	**(%)**	**N**	**(%)**	**N**	**(%)**	**N**
**Male**	1 (Basic)	215,607	(32)	120,082	(24)	79,745	(30)	53,249	(42)	25,022	(43)	131	(1)	*493,837*
	2	229,083	(34)	210,144	(42)	108,985	(41)	46,910	(37)	13,966	(24)	0	(0)	*609,087*
	3	134,755	(20)	50,034	(10)	15,949	(6)	5,071	(4)	1,746	(3)	0	(0)	*207,555*
	4 (High)	80,853	(12)	95,065	(19)	50,505	(19)	17,750	(14)	6,401	(11)	262	(2)	*250,836*
	Missing	20,213	(3)	25,017	(5)	10,633	(4)	2,536	(2)	10,474	(18)	12,722	(97)	*81,594*
	**All**	**673,773**		**500,343**		**265,816**		**126,784**		**58,190**		**13,115**		**1,638,021**
**Female**	1 (Basic)	171,753	(27)	141,319	(29)	109,669	(40)	91,760	(59)	52,508	(55)	320	(1)	*567,329*
	2	190,837	(30)	160,811	(33)	95,961	(35)	40,437	(26)	15,275	(16)	0	(0)	*503,320*
	3	146,308	(23)	38,984	(8)	13,709	(5)	4,666	(3)	1,909	(2)	0	(0)	*205,576*
	4 (High)	114,502	(18)	126,700	(26)	46,609	(17)	17,108	(11)	5,728	(6)	0	(0)	*310,647*
	Missing	19,084	(3)	24,365	(5)	8,225	(3)	3,111	(2)	20,048	(21)	31,359	(98)	*106,192*
	**All**	**636,122**		**487,306**		**274,173**		**155,525**		**95,469**		**31,999**		**1,680,594**
**Both**	**Total**	**1,309,895**		**987,646**		**539,989**		**822,298**		**101,359**		**45,114**		**3,318,615**

Danish residents without register markers of CVD, diabetes and/or statin treatment.

a) Educational levels:

1) 7–10 years basic school, 2) Vocational training after basic school, 3) High school or 9/10 years basic school with 1 year higher education 4) More than two years higher education.

From the Danish National Patient Registry, we retrieved information on patient discharge from non-psychiatric hospitals since 1977. Records include the admission and discharge dates, discharge diagnoses according to the International Classification of Diseases (ICD), 8^th^ revision until 1993, and 10^th^ revision thereafter (9^th^ revision has not been used in Denmark) along with codes for diagnostic and surgical procedures. We included main and secondary diagnoses for admitted patients and patients in ambulatory care. From the Registry of Causes of Death, we retrieved date and cause of death (primary and contributory).

Information on dispensed prescription drugs was retrieved from the Danish National Prescription Registry (DNPR), containing full information since 1996 on all out-of-hospital purchases of prescription drugs at Danish pharmacies including those of nursing home residents [43]. Records include the person identifier, date of dispensing, and the Anatomical Therapeutic Chemical (ATC) classification code of the dispensed drug [44]. From the DNPR we retrieved information on dispensed cardiovascular drugs (ATC groups C and B01) and antidiabetics (A10).

To identify asymptomatic individuals, we applied historical register data on in/out-patient diagnoses and procedures along with dispensed prescription drugs as register-markers for a range of CVD conditions, including ischemic heart disease (IHD) with or without myocardial infarction (MI), stroke, a range of other atherosclerotic conditions, and diabetes. We define asymptomatic individuals as individuals without register-markers of CVD or diabetes, as defined in a recent publication [45].

### Study design

While measures such as the Gini-coefficient of inequality, concentration index and the slope index of inequalities provide means for quantifying the degree of for example income-related inequality in health or health care delivery [46], a measure combining potential inequalities both in health care delivery and health care needs is indispensable to quantify inequities in health care delivery – if needs also are unequal across strata. However, measuring the need for preventive health care is a challenge, as such needs not may be captured by for example self-rated health scales. We opted to apply a need-proxy analogous to the underlying presumption of the risk-score chart, namely a measure of CVD- incidence in the background population of asymptomatic individuals, i.e. without CVD, diabetes or statin therapy - stratified by gender, 5-year age groups and SEP indicator.

Due to the high validity of the diagnosis of MI in the Danish registries [47], we applied the incidence of MI as need-proxy, using two alternative need-proxies in a sensitivity analysis: first stroke or MI as combined CVD endpoint and CVD as cause of death (MI, stroke or aortic aneurysm). Stratum specific MI-incidence rates were calculated, corresponding to number of incident MI cases (hospitalised or fatal) per 10,000 person-years at risk (PYR) during 2002–2006, censoring at death, emigration and register-markers of CVD, diabetes or statin therapy. Analogously, we calculated the ‘observed’ incidence of statin therapy (number redeeming the first statin prescription/10,000 PYR – censoring at death, emigration and register-markers of CVD or diabetes) and the combined MI-stroke endpoint. In order not to confine CVD-mortality to sudden CVD-death, CVD-mortality was calculated without censoring for new events of CVD or diabetes, covering also a longer span of time (2002–2008).

We applied a fixed SEP level corresponding to the beginning of the observation period (primo 2002). In order to capture income fluctuations over time, we calculated the average annual income between 1996 and 2001 (inflated to 2000 purchasing power), divided into income quintiles within gender and age group. The highest attained educational levels as of 2002 were divided into four groups according to length of formal education, cf. Table 1. As the analysis covers a time span of five years, individuals were considered to belong to a fixed 5-year age-group (i.e., that of 2002).

To evaluate horizontal inequity in initiation of preventive statin therapy, we adjusted the observed incidence of statin therapy according to the different needs across SEP-groups, applying stratum specific MI-incidence as proxy for needs. By means of indirect standardisation, we calculated the expected incidence of statin therapy (‘need-standardized statin incidence’), assuming that incidence of statin therapy must increase proportionally to the need across SEP-groups - for equity to be met. The need-standardized statin incidence was calculated as the observed statin incidence divided by the stratum specific need weights corresponding to the incidence rate ratio of MI (MI-IRR), Table 2. The denominator (PYR) of the observed statin incidence rather than the nominator (number of events) was need-standardized, dividing the observed PYR by MI-IRR.

**Table 2:** Observed and need-standardized incidence of statin treatment among asymptomatic individuals, according to gender, age-group and SEP indicators

**Gender**	**SEP**	**40-54**			**55-64**			**65-74**			**75-84**		
		**Need**	**Statin incidence**^**d**^		**Need**	**Statin incidence**		**Need**	**Statin incidence**		**Need**	**Statin incidence**	
	**INCOME**^**a**^	**Weight**^**c**^	**Observed**	**St.dized**^**e**^	**Weight**	**Observed**	**St.dized**	**weight**	**Observed**	**St.dized**	**weight**	**Observed**	**St.dized**
Male	1 (Low)	1.00	8.0	8.0	1.00	16.0	16.0	1.00	18.6	18.6	1.00	10.2	10.2
	2	0.90	9.0	10.0	1.01	19.1	18.9	0.96	22.2	23.2	0.81	10.9	13.5
	3	0.91	9.8	10.7	0.90	20.3	22.5	0.89	23.3	26.1	0.85	11.2	13.2
	4	0.82	10.2	12.4	0.80	21.2	26.4	0.79	23.2	29.3	0.77	12.6	16.3
	5 (High)	0.71	10.5	14.9	0.70	21.3	30.5	0.65	23.4	35.8	0.66	15.0	22.8
Female	1 (Low)	1.00	8.3	8.3	1.00	23.5	23.5	1.00	28.2	28.2	1.00	14.1	14.1
	2	0.80	8.3	10.4	0.85	25.2	29.8	0.85	29.4	34.6	0.90	15.3	17.1
	3	0.64	8.4	13.1	0.64	24.7	38.8	0.90	28.8	32.0	1.02	15.6	15.3
	4	0.60	8.5	14.1	0.57	23.1	40.5	0.69	29.2	42.0	0.82	17.7	21.6
	5 (High)	0.49	7.6	15.6	0.31	20.7	67.0	0.44	28.2	63.4	0.64	17.5	27.5
	**EDUCATION**^**b**^												
Male	1 (Basic)	1.00	9.4	9.4	1.00	18.7	18.7	1.00	20.5	20.5	1.00	11.6	11.6
	2	0.81	10.2	12.6	0.89	20.6	23.2	0.97	23.9	24.6	0.94	14.6	15.6
	3	0.59	8.3	14.1	0.75	18.5	24.6	0.82	22.1	27.0	0.76	13.9	18.2
	4 (High)	0.50	8.8	17.8	0.66	19.9	30.2	0.65	23.8	36.7	0.72	15.7	21.8
Female	1 (Basic	1.00	9.9	9.9	1.00	25.6	25.6	1.00	28.9	28.9	1.00	17.3	17.3
	2	0.63	8.8	14.0	0.68	23.8	35.2	0.79	30.0	38.1	0.82	20.2	24.7
	3	0.43	6.0	14.0	0.47	18.7	39.6	0.54	25.2	46.3	0.68	17.2	25.3
	4 (High)	0.34	6.2	18.2	0.38	19.1	50.8	0.53	26.4	50.2	0.65	19.0	29.5

a) Quintiles of disposable family income within gender and 5-years age-groups – inflated average over 1996–2001.

b) Educational levels, cf. notes in Table 1.

c) Incidence Rate Ratio of myocardial infarction (MI-IRR) among asymptomatic individuals, applying lowest SEP group (within gender and age-group) as reference group.

d) Incidence of statin therapy among asymptomatic individuals: 1000*(Number incident statin dispensing/person years at risk).

e) Need-standardized statin incidence (‘St.dized’): Observed statin incidence/MI-IRR.

**Figure 1:**
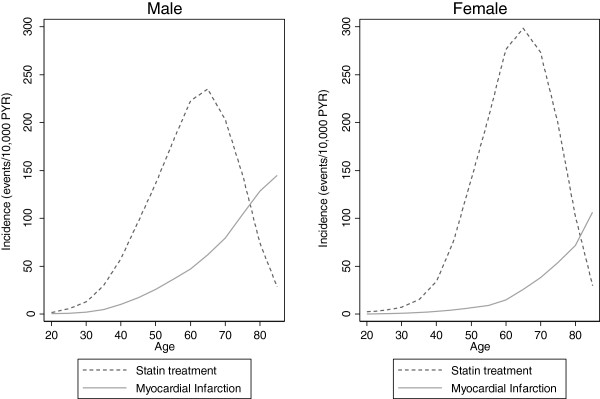
Incidence of statin therapy versus incidence of myocardial infarction during 2002–2006 in asymptomatic individuals.

Based on the need-standardized statin incidence parameters (observed statin events (counts), need-standardized PYR (exposure)), Poisson regression analyses were applied to test the overall horizontal equity across SEP. With the lowest SEP-group (and age group) as reference, a need-standardized statin-IRR > 1 translates into horizontal inequity favouring the higher SEP-groups. The null hypothesis, horizontal equity, corresponds thus to statin-IRR = 1. We estimated a *‘horizontal inequity gradient’* (the *overall* linear trend) reflecting the increase in need-standardized statin-IRR for each increase in SEP*.* Owing to a gender and age specific pattern of both MI-incidence (need) and incidence of preventive statin therapy, we stratified the analyses according to gender and ages +/−65, cf. Figure 1.

Nonparametric bootstrapping [48] was applied to incorporate the precision of the need-weights in the confidence intervals (CI) of the need-standardized statin IRR. Based on 10,000 bootstrap replications, need-weights were calculated and applied in the Poisson regression analyses of need-standardized statin incidence parameters. Normal based 95% CI from the bootstrapping procedure were applied as CI for the point estimate for statin IRR calculated from the original data.

All analyses were performed using Stata Release 11.1 (StataCorp, College Station, TX, USA). Access to data was provided and secured through collaboration between the University of Copenhagen and Statistics Denmark. Register-based studies in Denmark do not require approval by an ethics board.

## Results

Figure 1 depicts the incidence of MI and statin therapy among asymptomatic individuals during 2002–2006 according to age, stratified by gender. Both in men and women, the MI-incidence increases gradually with age, whereas statin incidence increases steeply until the age of 65, decreasing markedly thereafter. While the MI-incidence is highest among men of all ages, the opposite is the case as regards statin incidence.

Table 2 shows that the need-weights (MI-IRR relative to the lowest SEP-group) are decreasing with increasing SEP – independently of gender and age categories. In men aged 55–64, the need (MI-incidence) in the highest income quintile is 70% of that in the lowest income quintile, in women the figure is 30%. Above the age of 75 the gradient is less pronounced. Analogously, when applying four educational levels as an indicator for SEP, the need in men aged 55–64 with the highest educational level is 70% of those with basic education – in women the figure is 40%. Yet, while the observed statin incidence increases with increasing income in men, only, the need-standardized statin incidence increases steeply with increasing income in both genders – and more so among women due to the steeper gradient in MI-incidence.

Table 3 presents the results of the gender/age stratified Poisson regression analyses on need-standardized statin parameters. In men aged 40–64, the need-standardized statin incidence increases by 17% (IRR 1.17; 95% CI 1.14-1.19) for each increase in income quintile – corresponding to the horizontal inequity gradient (HIE-gradient). In women the HIE gradient is greater 23% (1.23; 1.16-1.29). Among individuals older than 65, the corresponding HIE-gradient is 17% (1.17; 1.14-1.20) and 20% (1.20; 1.14-1.27), in men and women, respectively.

**Table 3:** Result of Poisson regression analysis, testing horizontal equity across levels of socio-economic position (SEP) with differential needs

**SEP indicator**^** *a* **^	**Age 40-64**				**Age 65-84**			
	**Male**		**Female**		**Male**		**Female**	
**Income Quintiles**	**IRR**^**b**^	**95% CI**^** *c* **^	**IRR**	**95% CI**	**IRR**	**95% CI**	**IRR**	**95% CI**
1	1.00		1.00		1.00		1.00	
2	1.22	(1.10-1.35)		1.27	(1.04-1.55)	1.27		(1.05-1.54)	1.25	(1.04-1.51)
3	1.38	(1.29-1.48)		1.63	(1.39-1.92)	1.38		(1.17-1.63)	1.16	(0.93-1.45)
4	1.61	(1.47-1.76)		1.73	(1.40-2.15)	1.58		(1.36-1.84)	1.54	(1.27-1.86)
5	1.89	(1.70-2.10)		2.44	(1.86-3.21)	1.99		(1.68-2.35)	2.26	(1.80-2.80)
*HIE gradient*^*d*^	*1.17*	*(1.14-1.19)*		*1.23*	*(1.16-1.29)*	*1.17*		*(1.14-1.20)*	*1.20*	*(1.14-1.27)*
**Educational level**	**IRR**	**95% CI**	**IRR**	**95% CI**	**IRR**	**95% CI**	**IRR**	**95% CI**		
1	1.00		1.00		1.00		1.00			
2	1.29	(1.17-1.42)		1.38	(1.27-1.49)	1.19		(1.07-1.33)	1.33	(1.13-1.56)
3	1.40	(1.18-1.66)		1.49	(1.33-1.65)	1.34		(1.19-1.52)	1.52	(1.23-1.89)
4	1.74	(1.51-2.01)		1.90	(1.72-2.09)	1.77		(1.59-1.97)	1.70	(1.47-1.97)
HIE gradient	*1.19*	*(1.15-1.24)*		*1.24*	*(1.19-1.29)*	*1.21*		*(1.17-1.25)*	*1.21*	*(1.14-1.28)*

a) Indicators of SEP: Quintiles of disposable income and 4 levels of formal education, cf. notes Table 1.

b) Poisson regression analyses of need-standardized statin Incidence Rate Ratio (IRR), applying need-standardized statin incidence parameters within gender and 5-year age-groups, based on indirect standardization with Incidence of myocardial infarction in the background population as need-weights, cf. Table 2.

c) Bootstrapping (10,000 reps.) is applied for 95% confidence intervals (CI).

d) Horizontal Inequity (HIE) gradient: The relative change in need-standardized statin incidence for each increase in SEP, i.e., the estimated overall linear trend.

The HIE-gradient concerning educational level reveals a similar pattern, i.e., 19% (1.19; 1.15-1.24) in men aged 30–64, and 24% (1.24; 1.19-1.29) in women.

Table 4 shows sensitivity analysis of the need-proxy, where the HIE-gradient is calculated applying the original need-proxy, MI incidence, as well as the two alternative need-proxies: MI/stroke as combined end-point and CVD as cause of death, respectively. Independently of need-weights (need-proxy) used, the sensitivity analysis revealed the same pattern of horizontal inequity, favouring the better-off. Yet, the HIE-gradient is attenuated when applying MI/stroke as a combined end-point. This is particularly true for women. Conversely, the HIE-gradient is enhanced when applying CVD-death as need-proxy – especially among men.

**Table 4:** **Sensitivity analysis of horizontal equity in incidence of preventive statin therapy: Three alternative need-weights (need-proxies) in the need-standardized analyses**^**a**^**: Myocardial infarction (MI), combined MI-stroke or CVD-death**^**b**^

**SEP indicator**^**c**^	**Age**	**Horizontal Inequity gradient (IRR)**^**d**^**dependent on need-weights**					
		**MI**		**MI-stroke**		**CVD-death**	
		**Male**	**Female**	**Male**	**Female**	**Male**	**Female**
**Income**	40-64	1.17	1.23	1.18	1.15	1.39	1.36
	65-84	1.17	1.20	1.15	1.10	1.29	1.23
**Educational level**	40-64	1.19	1.24	1.18	1.11	1.33	1.22
	65-84	1.21	1.21	1.15	1.11	1.23	1.29

a) Need-standardized Poisson analyses see notes Table 3.

b) Myocardial infarction (MI) as need-weight in the basic analyses. Two alternative CVD need: Incidence of MI and stroke as combined endpoint during 2002–2006, and CVD-death (i.e., MI, stroke and aorta-aneurism) during 2002–2008.

c) Indicators of socio-economic position (SEP): cf. notes Tables 1 & 3.

d) Horizontal Inequity (HIE) gradient: The relative change in need-standardized statin incidence for each increase in SEP, i.e., the estimated overall linear trend.

## Discussion

### Principal findings

Applying indirect standardisation and MI-incidence as a proxy for need, we developed a pharmacoepidemiological method to explore horizontal equity in initiation of preventive statin therapy across SEP-groups with unequal needs, adjusting the observed statin incidence according to relative needs across socio-demographic groups. Our study indicates that the high-risk strategy (as implemented in Denmark) to prevent CVD by initiating preventive statin therapy is inequitable, reaching primarily high-risk individuals in low-risk SEP-groups. The favouring of more advantaged groups holds for both genders, independently of applying income quintiles or educational level as SEP indicator. In men aged 30–64, the need-standardized statin incidence increased by 17% for each increase income quintile (the horizontal inequity gradient) – in women the increase was 21%.

Only among men, the *observed* incidence statin therapy tended to increase with increasing SEP, but due to a steeper social gradient in MI-incidence among women, the horizontal inequity gradient of initiating preventive statin therapy was steepest in women.

### Strengths and limitations

Given the inverse relationship between SEP and CVD, the challenge of this study examining equity in the medicamental high-risk strategy to prevent CVD was twofold: firstly, to operationalize need and equity in CVD preventive drug therapy across SEP-groups with unequal needs, and secondly, to develop appropriate pharmacoepidemiological methods for testing horizontal equity.

### Needs

We opted to apply nationwide register data on MI-incidence in the statin free and asymptomatic background population stratified by gender, age and SEP as need-proxy, instead of calculating individual-level CVD-risk based on survey information on CVD-risk factors and risk scoring,

This is a strength for at least three reasons:

1) The nationwide approach is without the well-known selection bias problems from cohort studies/surveys where people are invited to participate.

2) The risk-score charts generally have low predictive value – both at the individual and the group level. Various cohort studies indicate that standard risk-score charts tend to underestimate CVD-risk in worse-off groups, overestimating the risk in better-off groups [36, 49, 50], and attempts have been made to modify risk-score charts according to the actual background populations [51] and/or socioeconomic groups [49, 50]. Yet, individual risk prediction is notoriously difficult [52, 53], and as most CVD events occur in people with modest risk-factor values, overlapping with those seen in people without CVD, the appropriateness of applying individual risk-factor levels as a screening tool has been questioned [52, 54, 55]. The risk-score charts applied in Denmark seems, in fact, to have a very poor predictive value [56]. In the present study we applied risk (MI-incidence) at the sub-group level (gender, age and SEP) as need-proxy rather than risk at the individual level. Yet, individual risk estimates based on risk-scoring relies on risk calculated at the group level (age and gender) from historic survey data.

3) Register-based information on MI-diagnoses is regarded as valid in-hospital diagnosis information [47]. Thus, the estimated MI-incidences should reveal the actual SEP gradient in MI-incidence. However, due to potential differential symptom awareness and communication barriers, a social gradient in admission for MI may exist, leading to an underestimated MI-incidence in less advantaged groups. The better-off, on the other hand, may be more likely to prevent MI by means of invasive coronary procedures, leading to a potential underestimated MI-incidence here. Yet, including acute invasive coronary procedures as marker for MI revealed the same results (data not shown). Thus, we regard this bias of minor importance. In a sensitivity analysis, stroke/MI as a combined endpoint and CVD-death were tested as two alternative need-proxies. Independently of need-measure, the sensitivity analysis demonstrated similar patterns of horizontal inequity. Yet, when applying the combined endpoint, the horizontal inequity gradient was – especially in women – attenuated, presumably due to a less pronounced social gradient in the incidence of stroke than in MI-incidence. Applying CVD-death as a need-proxy (corresponding to the risk measure applied in Danish risk-scoring charts), the horizontal inequity was enhanced. Yet the ‘validity’ of actual MI-incidence as the need-proxy rests upon the assumption that the need for statin therapy is a question of CVD-risk rather than a single risk-factor level – and partly on the finding that conventional risk-factors to a large extent mediate the social gradient in CVD-risk (in absolute terms). However, high cholesterol level has not consistently been found to increase with decreasing SEP [6, 8, 57], potentially explained by the rather imprecise cholesterol parameters applied: risk thresholds for cholesterol levels and measurement of total cholesterol without distinguishing between the various lipid-fractions. Conversely, other studies have demonstrated an association between the metabolic syndrome (caused by physical inactivity, unhealthy diets, and obesity) and elevated low-density lipid cholesterol (LDL) [58], indicating that the inverse social gradient in LDL may follow the social stratification in physical inactivity and obesity.

### Pharmacoepidemiological method for testing equity

Analogous to studies within economic equity research [19,22,42], we applied indirect standardisation to evaluate horizontal equity in health care delivery. In a study on equity in US ambulatory care [42], the number of ambulatory visits was adjusted according to differential self-rated health. Applying the need-standardized counts of the dependent variable (ambulatory visits) and a continuous income variable as the explanatory variable, a ‘*horizontal inequity index’* (i.e., a need-standardized concentration index) was estimated. In our pharmacoepidemiological approach, we calculated, instead, a need-standardized incidence rate of statin therapy. Applying need-standardized statin incidence parameters as a dependent variable and a SEP indicator as an ordinal explanatory variable (e.g., income quintiles), we estimated a *horizontal inequity gradient*. We consider this methodological analogy to be a strength. Yet, while it is intuitively reasonable to ‘adjust’ for differential health conditions when evaluating horizontal equity in ambulatory visits, it may be less obvious that incidence of preventive CVD-drug therapy should be proportional to the risk of disease for equity to be met.

### Interpretation and comparison with other studies

While a range studies have demonstrated *inequality* in prescription of CVD preventive drugs [23,59-61], no studies have examined and quantified inequities, including both prescribing patterns and needs in a nationwide perspective. In contrast to a Norwegian health survey study [40] showing a decreasing trend of incidence of statin treatment by increasing education in individuals without reported CVD or diabetes at baseline, we found almost the same incidence across educational groups among asymptomatic individuals, censoring for new onset of CVD/diabetes. The lack of censoring for onset of disease in the Norwegian study most likely explains the discrepancy between the studies, as lower SEP individuals are at higher risk of developing disease and may thereby be misclassified as free of CVD or diabetes when initiating statin treatment. Our finding that the high-risk strategy as implemented in Denmark seems to be inequitable may reflect both the poor predictive value of the applied risk-score charts and a selective uptake. The latter being an inherent consequence of applying an opportunistic screening strategy, where uptake depends on the client’s participation and the physician’s general judgement of her/his client. A so-called ‘healthy user effect’ has been shown in pharmacoepidemiological studies, indicating that preventive measures (e.g., vaccinations and drugs) tend to be used by population segments with a broad spectrum of healthier behaviours [62]. With the consistently shown social gradient in CVD in most Western countries, our findings are likely to be applicable in other settings applying an opportunistic screening strategy. Several studies have demonstrated a socioeconomic gradient in screening uptake [63], indicating both financial (due to drug co-payment) and psychosocial barriers in socially deprived groups [64]. Psychosocial barriers to CVD screening may include negative perceptions about screening tests, risk perceptions and the social stress associated with talking about unhealthy lifestyles with the GP – of higher SEP. Our findings may also reflect that high CVD risk in lower SEP at first hand is ‘attacked’ by encouraging individual lifestyle modifications.

In line with other studies [61,65] our study indicates that the high-risk strategy may widen the socioeconomic gradient in CVD owing to the inequitable uptake. However, any widening of the CVD incidence gradient depends on the outcome of therapy and not merely on initiation of therapy. Here two other issues are important: Differential adherence to therapy and differential outcome of therapy. In fact, long-term adherence to statin treatment is disappointing [66] – and is likely to depend on SEP, indication and experienced adverse effects (potentially weighted against the perceived beneficial effect). While the risk of life-threatening adverse effects is low (e.g., rhabdomyolysis), various degrees of muscle side effects are not unusual, ranging from muscle weakness to rhabdomyolysis [67]. If both incidence and duration of therapy are lowest among less advantaged groups – the social gradient in prevalence and outcome of therapy is likely to be even steeper than the gradient found as to initiation of therapy. However, being exposed to multiple risk factors (known and unknown) acting in concert, socially disadvantaged groups may be more vulnerable to high LDL levels than the better-off. Hence, the *outcome* or beneficial effect of lifelong preventive statin therapy may be greater in less advantaged groups – provided adherence to therapy.

### Unanswered questions and future research

Various incentives have been proposed to enhance adherence, requiring often GPs to be more actively involved [68,69]. In a forthcoming study on the same nationwide Danish data we explore potential socioeconomic differences in adherence to statin treatment in asymptomatic individuals.

The incidence of preventive statin treatment in this study was found to peak around the age of 65, and to decrease steeply hereafter. This pattern may reflect the widespread use of the risk-score charts, covering the age range of 40–65, potentially representing an issue of ageism. The finding that statin incidence is considerably higher in asymptomatic women than men although MI-incidence is higher in men (cf. Figure 1) may both reflect a consequence of an opportunistic preventive screening strategy and an overestimation of CVD-risk in Danish women, corresponding to the finding in a Norwegian study [70]. Both matters will require further research.

In contrasts to the opportunistic screening strategy applied in Denmark, a universal screening programme to prevent CVD is actually being implemented in the UK [71]. Here, all asymptomatic individuals aged 40–74 are invited for risk-scoring and potential preventive statin therapy. Yet, there is an ongoing discussion about the programme covering both resource implications [72] and the dilemma between a targeted high-risk strategy with a high CVD risk threshold for initiating therapy with potentially non-generic more potent and effective statins versus a “population strategy” in which all invited individuals should be prescribed low-price preventive treatment (generic statins) [71] or eventually a Polypill (a combination of four CVD-preventive drug categories) [73,74],bypassing the risk-scoring charts of poor predicative value and potentially also subsequent GP visits. While recent cost-effectiveness reviews indicate a very high cost effectiveness of Polypill strategies [75], a pilot project may uncover equity concerns before implementation of a general screening strategy. Another strategy could be to focus the high-risk drug strategy on middle-aged asymptomatic men in whom the beneficial effect of preventive statin treatment is best documented, testing various settings in order to reach lower SEP groups before implementation - potentially also adjusting the reimbursement system accordingly. Yet, by not controlling the causes of high CVD incidence (e.g., low cigarette prices; high levels of saturated and *trans*-fats, sugars, salt hidden in processed foods, and environmental factors) this ‘population’ strategy will be palliative and not radical as structural/population strategies tends to be [55]. Proposing a range of actions to be taking, a newly published Danish report “Health inequality - determinants and policies” [76] demonstrates that reducing health inequality is not primarily a health care task, but a complex task requiring coordinated efforts from different sectors (e.g. the education, social, healthcare, and employment sectors).

## Conclusions and implications for policy and practice

Our study indicates that the high-risk strategy to prevent CVD by means of preventive statin therapy – as practiced in Denmark – is inequitable, primarily reaching high-risk individuals in low-risk groups, i.e., individuals in higher SEP-groups. The inequity is likely to be the consequence of using a screening tool with low predictive value and a screening programme with differential socioeconomic uptake. Provided long-term adherence and a beneficial effect of preventive statin therapy independent of SEP, the strategy may contribute to accentuating the inverse relationship between SEP and CVD. Facing the challenges posed by an ageing population, one might question to what extent scarce GP resources should be allocated for better-off, asymptomatic individuals.

## Abbreviations

SEP, Socioeconomic position; CVD, Cardiovascular disease; RCTs, Randomised clinical trials; GP, General practitioner; DNPR, Danish National Prescription Registry; ICD, International Classification of Diseases; ATC, Anatomical Therapeutic Chemical classification system; IHD, Ischemic heart disease; MI, Myocardial infarction; PYR, Person-years at risk; IRR, Incidence rate ratio; HIE gradient, Horizontal inequity gradient.

## Competing interests

The authors declare no conflicts of interest. Morten Andersen participates, however, in a research project on statins funded by AstraZeneca, Merck Sharp & Dohme and Pfizer.

## Authors’ contribution

All authors read and approved the final manuscript.

## Authors’ information

Finn Diderichsen, Allan Krasnik and Theis Lange contributed equally to the work.

Helle Wallach-Kildemoes, first and corresponding author.

Morten Andersen contributed as last author and principal co-author.

## Pre-publication history

The pre-publication history for this paper can be accessed here:

http://www.biomedcentral.com/1471-2458/12/610/prepub
